# Detecting β-Casein Variation in Bovine Milk

**DOI:** 10.3390/molecules21020141

**Published:** 2016-01-25

**Authors:** Anna Maria Caroli, Salvatore Savino, Omar Bulgari, Eugenio Monti

**Affiliations:** Unit of Biotechnology, Department of Molecular and Translational Medicine, University of Brescia, Brescia 25123, Italy; salvatore.savino@unibs.it (S.S.); omar.bulgari@unibs.it (O.B.); eugenio.monti@unibs.it (E.M.)

**Keywords:** bovine milk, β-casein, bioactive peptide, isoelectric focusing

## Abstract

In bovine species, β-casein (β-CN) is characterized by genetic polymorphism. The two most common protein variants are β-CN A^2^ (the original one) and A^1^, differing from A^2^ for one amino acid substitution (Pro_67_ to His_67_). Several bioactive peptides affecting milk nutritional properties can originate from β-CN. Among them, β-casomorphin-7 (BCM7) ranging from amino acid 60 to 66 can be released more easily from β-CN variants carrying His_67_ (A^1^ type) instead of Pro_67_ (A^2^ type). Nowadays, “A2 milk” is produced in different countries claiming its potential benefits in human health. The aim of this study was to further develop and apply an isoelectric focusing electrophoresis (IEF) method to bulk and individual milk samples in order to improve its use for β-CN studies. We succeeded in identifying A2 milk samples correctly and quantifying the percentage of A^2^, A^1^, and B variants in bulk samples not derived from A2 milk as well as in individual milk samples. The method allows us to quantify the relative proportion of β-CN variants in whole milk without eliminating whey protein by acid or enzymatic precipitation of caseins. The aim of this study was also to study the different behavior of β-CN and β-lactoglobulin (β-LG) in the presence of trichloroacetic acid (TCA). The higher sensitivity of β-CN to TCA allows quantifying β-CN variants after TCA fixation because β-LG is not visible. Monitoring β-CN variation in cattle breeds is important in order to maintain a certain balance between Pro_67_ and His_67_ in dairy products. Overall, the debate between A1 and A2 milk needs further investigation.

## 1. Introduction

**Figure 1:**
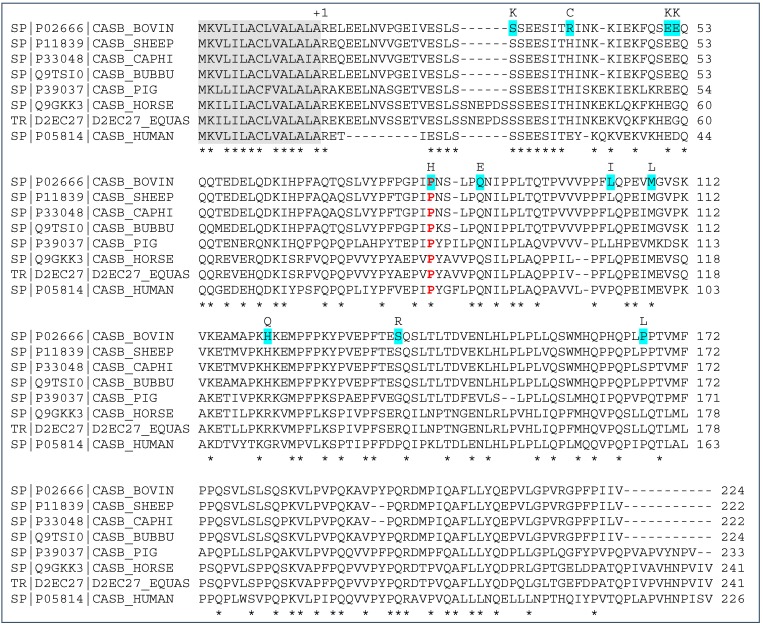
Multiple alignment of β-CN pre-protein in different species, in the following order: cattle, sheep, goat, buffalo, pig, horse, donkey, and human. P02666 sequence corresponds to the bovine A^2^ variant. Outlined in grey: signal peptide; in light blue: natural variations in cattle (the substituted amino acid is written above); in red: the amino acid corresponding to Pro_67_ within the bovine mature protein sequence. +1: start of the mature protein. Symbol *: no amino acid substitution.

In bovine species, β-casein (β-CN) accounts for 9–11 g/L of skim milk and is characterized by genetic polymorphism [1]. The two main protein variants are β-CN A^2^ and A^1^, differing by one amino acid substitution (Pro_67_ to His_67_) [2]. Overall, a total of 12 protein variants were described. The original β-CN variant carries Pro_67_, as highlighted by the comparison among cattle, sheep, goat, buffalo, pig, horse, donkey, and human pre-proteins (Figure 1). The bovine sequence refers to β-CN A^2^ which is the original variant within the *Bos* genus. The bovine β-CN variants carrying Pro_67_ are A^2^, A^3^, D, E, H^2^, and I, while His_67_ occurs in A^1^, B, C, F, G; this information is not available for H^1^ [2]. In taurine breeds, A^2^, A^1^ and B are common variants, and C and I are rather common, whereas the remaining variants are rare and do not usually occur [2].

Bioactive peptides are protein fragments that can be released by enzymatic cleavage during protein digestion or food processing [3,4]. They can exert favorable effects on human health [5]. A wide variety of bioactive peptides affecting milk nutritional properties can originate from caseins [6,7]. Biopeptides might be affected by amino acid substitutions or deletions resulting from genetic polymorphism [2]. Thus, β-casomorphin-7 (BCM7), ranging from amino acid 60 to 66 of mature β-CN, has opioid properties and can be released much more easily from β-CN variants carrying His_67_ (A^1^-type variants) instead of Pro_67_ (A^2^-type variants) [8,9]. Different negative effects have been suggested for the consumption of the so-called “A1 milk” [2]. However, the European Food Safety Authority (EFSA) concluded that a cause-effect relationship cannot be established between the oral intake of BCM7 or related peptides and the etiology of any suggested diseases [10]. Therefore, EFSA did not recommend any formal risk linked to such food-derived peptides. In all cases, nowadays “A2 milk” is produced in different countries, and further studies have been carried out on the disadvantages of A1 milk, mainly pointing out its potential negative effects on human digestive function [11,12,13,14].

Different methods are available for the separation and quantification of bovine milk protein variants by reversed phase high-performance liquid chromatography (RP-HPLC) [15] or high resolution HPLC-mass spectrometry (HPLC-MS) [16]. Recently, a test was developed aimed at quantifying the relative percentage of A2 β-CN of total β-CN at the level of whole bulk milk based on the use of isoelectric focusing (IEF) [17]. The IEF technique is followed by the acquisition of the gel after fixation in trichloroacetic acid (TCA) and succeeding quantification of β-CN–related bands with a simple software for gel analysis.

The aim of this study was to study the different behavior of β-CN and β-lactoglobulin (β-LG) in the presence of TCA. This difference allows quantifying β-CN variants immediately after TCA fixation because β-LG overlapping, which occurs if the IEF gel is stained with Coomassie Blue, is avoided. Moreover, this IEF method was improved and applied to bulk and individual milk samples in order to further develop its potential use both for β-CN genetic studies and practical applications.

## 2. Results and Discussion

### 2.1. Comparison between β-CN and β-LG Behavior in the Presence of TCA

**Figure 2:**
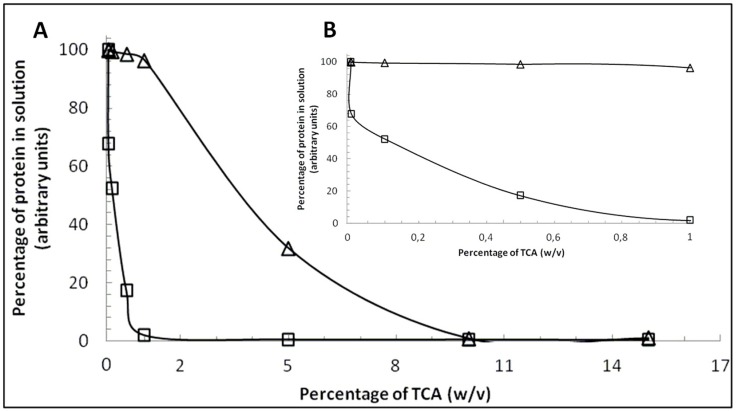
Panel (**A**): The percentage of protein in solution β-LG (open triangle) and β-CN (open square) after TCA-induced precipitation, expressed as *w*/*v*; Panel (**B**): Magnification of protein behavior at percentage of TCA from 0 to 1 (*w*/*v*).

The different behavior of β-CN and β-LG in the presence of TCA was investigated by a colorimetric assay to determine the concentration of the two standard proteins in different TCA solutions. The percentage of protein in solution with β-LG and β-CN after TCA-induced precipitation is shown in Figure 2. The TCA precipitation curves show a different behavior depending on the milk protein. Briefly, β-CN is highly sensitive to acid precipitation, as expected from TCA treatment of IEF gels, whereas β-LG is more resistant to TCA precipitation. In the solution conditions applied during the colorimetric assay, both proteins precipitate completely at 10% TCA concentration (Figure 2).

The amount of β-LG and β-CN in bovine milk has been quantified using RP-HPLC and corresponds to 5.02–5.77 g/L and 11.85–13.4 g/L, respectively [18]. By using capillary zone electrophoresis (CZE), the relative concentration of the six major milk proteins was estimated and β-LG and β-CN show a mean value of 8.35% and 27.17%, respectively [19]. Overall, the differences in the relative amount of β-LG and β-CN in milk, as well as the higher sensitivity of the latter to TCA precipitation, explain the staining differences of IEF gels when treated with TCA before the final staining with Coomassie Blue.

### 2.2. Content of β-CN in Bulk Milk Samples

**Figure 3:**
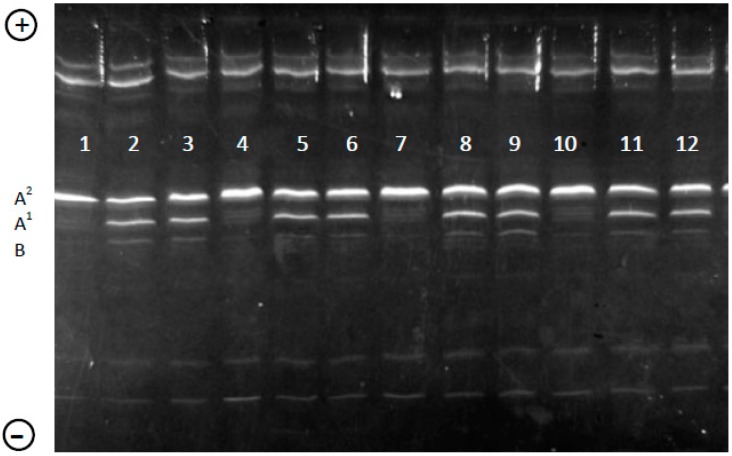
IEF analysis of bulk milk samples. Samples 1, 4, 7, and 10 are from A2 milk. The positions of the three most common β-CN variants (A^1^, A^2^ and B) are indicated on the left side of the gel. In the upper (+) and lower (−) part of the gel α_S1_-CN and κ-CN are visible, respectively.

The method applied allowed us to identify all A2 milk samples correctly (four A2 milk samples in a total of 20 samples) and to quantify the percentage of A^2^, A^1^, and B variants in the bulk samples not derived from pooled milks obtained only from known A^2^ individual milk samples (Figure 3).

Quantitation of the protein band content showed that in the “not A2” samples the highest β-CN proportion was found for A^2^, ranging from 60.5% to 64.8%, followed by A^1^ (24%–33.6%), and B (4.8%–13.3%). These ratios are in agreement with the distribution of the allele frequencies of the three most common β-CN variants in European bovine dairy breeds. As an example, we refer to Holstein Friesian which is the most common dairy breed, spread all over the world for its high milk yield. In Italian Friesian, the frequency of β-CN A^2^ plus I variants accounted for 0.570, followed by A^1^ (0.395), and B (0.035) [20]. It is necessary to point out that A^2^ and I variants comigrate in IEF, but these variants differ for one amino acid exchange (Met_93_ in A^2^ to Leu_93_ in I) not affecting Pro_67_, which is carried by both polypeptides [21]. In 2005, Dutch Holstein Friesian A^2^ plus I accounted for 0.692, A^1^ for 0.285, A^3^ for 0.001, and B for 0.022, showing an increase in the frequency of β-CN A^2^ at the expense of β-CN A^1^ compared to a previous study carried out in 1989 [19]. Conversely, in Italian Friesian no substantial modification of the frequency of β-CN variants was found in a 10-year period [20]. In Swedish Holstein, the frequencies of β-CN A^2^, β-CN A^1^, and B were 0.60, 0.34, and 0.06, respectively [18].

Quantifying IEF gels in TCA represents a great advantage because, after Coomassie Blue staining, β-LG overlaps the β-CN migration zone, making it difficult to distinguish between two variants of these proteins, *i.e*., β-LG*B and β-CN*A^1^. Acid or enzymatic precipitation of caseins before their analysis with IEF could be a solution for quantifying the gels after Coomassie Blue staining, but in ultra-high temperature (UHT) treated milk, β-LG denatures producing complexes with casein [22], thus precipitation does not allow eliminating β-LG with whey.

### 2.3. Genotyping of β-CN in Individual Milk Samples

**Figure 4:**
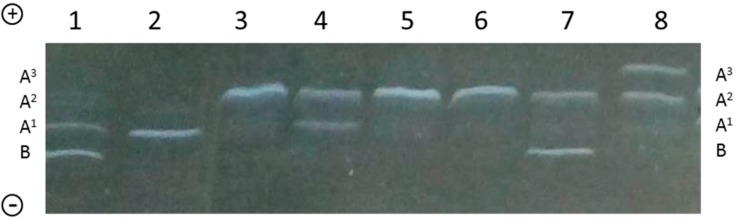
IEF analysis of individual milk samples from Italian Friesian cows. The β-CN variants are indicated on both gel sides. Sample 1: A^1^B; sample 2: A^1^A^1^; samples 3, 5, 6: A^2^A^2^; sample 4: A^1^A^2^; sample 7: A^2^B; sample 8: A^2^A^3^.

The IEF method, which is well known for the identification of the main β-CN variants in individual milk samples, is also efficient in quantifying their relative proportion in whole milk without eliminating whey protein by acid or enzymatic precipitation of caseins (Figure 4). Since the overlapping of whey proteins with β-CN is not detectable in TCA, the quantification of the relative amount of β-CN variants can be easily performed when monitoring β-CN protein variation by IEF in the different cattle breeds and populations.

A quantitative evaluation of the protein variants was also carried out in individual milk samples, allowing the detection of different allelic expression levels. A significantly higher percentage (+4.3%, *p* < 0.03) was found for the B variant with respect to A^1^ and A^2^ in the heterozygous genotypes A^1^B and A^2^B. This intriguing aspect, which recalls the different expression of other milk protein variants, e.g., β-LG*A > B, κ-CN*B > A [2], needs to be further investigated. The IEF method proposed here could be applied easily to monitor both qualitative and quantitative variation of β-CN in individual milk samples. It is important to point out that IEF allows the simultaneous detection of 28 of the 45 milk protein variants detected in *Bos taurus*, and 23 of them are not rare variants [23].

Monitoring of the distribution of β-CN variants of individual milk samples from different bovine breeds is important in order to maintain a certain balance between Pro_67_ and His_67_ in milk and dairy products, avoiding a possible excessive increase in His_67_ in the dairy breeds.

## 3. Experimental Section

Multiple alignment of β-CN pre-protein in different species was performed by CLUSTAL O (1.2.1) [24].

The different behavior of β-CN and β-LG in TCA was investigated by a colorimetric assay to determine the concentration of the two standard proteins in different TCA solutions (0% to 15%). Specifically, purified β-CN and β-LG from bovine milk (Sigma-Aldrich, St. Louis, MO, USA) were dissolved in deionized water and treated with TCA [25]. Protein solutions containing appropriate concentrations of the acid were prepared by the addition of requisite amounts of the stock solution of the acid to aqueous solutions of proteins. The TCA-treated protein solutions were incubated at 25 °C for 30 min. The precipitated protein samples were pelleted down by centrifugation at 12,000 rpm for 20 min at 25 °C. Protein concentrations in the supernatant were determined using the respective molar extinction coefficients of the proteins at 280 nm. All the UV spectrophotometric measurements were carried out on a GeneQuant 100 spectrophotometer (GE Life Sciences, Uppsala, Sweden).

The IEF method [26] was applied to both individual and bulk milk samples [17] in order to quantify the main variants of β-CN more effectively and, in particular, the A^1^ variant which migrates very close to the B variant of β-LG. IEF was carried out on ultrathin polyacrylamide gels (250 × 115 × 0.3 mm) with carrier ampholytes and using a GelBond^®^ PAG Film as a support. The screening gel with 8 M urea contained 0.15% (*w*/*v*) bisacrylamide, 4.49% (*w*/*v*) acrylamide, 1.4% (*v*/*v*) Pharmalyte (GE Healthcare Europe GmbH) pH 2.5–5, 2.8% (*v*/*v*) Pharmalyte pH 4.2–4.9, 2.3% (*v*/*v*) Pharmalyte pH 5–8. Samples contained 14% (*v*/*v*) whole milk, 40 *w*/*v* Urea and 2.2 *v*/*v* 2-β-mercaptoethanol. The IEF gels were acquired for imaging analysis immediately after fixation in TCA 15% (*w*/*v*) for 10 min. In fact, the bands related to β-CN are visible more clearly in TCA than those relating to the two main whey proteins. Gels were acquired and quantified using G:Box system (Syngene, model rating, Frederick, MD, USA).

A total of 20 bulk milk samples were examined, among which four were from A2 milk. Moreover, 40 individual milk samples from Italian Friesian were analyzed and quantified by the same method. The different expression of β-CN*B with respect to A^1^ and A^2^ variants was assessed by Student’s *t*-test.

## 4. Conclusions

The relatively cheap and rapid IEF protocol used in this study allows us to efficiently evaluate the incidence of the A2 milk in both bulk and individual milk samples. The protocol allowed us to study the distribution of type A2 β-CN variants in the different herds and production areas. This information is useful to avoid excessive reduction of the β-CN variants associated with this type of milk and, therefore, with a lower potential liberation of BCM7 from milk and derivatives. Moreover, from a commercial point of view the protocol should be important not only to highlight the presence of type A2 variants, but also to quantify their content in milk and dairy products not deriving from selected A2 cows.

The human health implication of A2/A1 milk is still an open debate that deserves further investigation and the proposed IEF protocol represents a useful tool for screening β-CN variants in both bulk and individual milk samples which could help in obtaining new data on this intriguing issue.
